# The clinical course of trauma-related disorders and personality disorders: study protocol of two-year follow-up based on structured interviews

**DOI:** 10.1186/s12888-017-1339-6

**Published:** 2017-05-10

**Authors:** Sanne Swart, Marleen Wildschut, Nel Draijer, Willemien Langeland, Jan H. Smit

**Affiliations:** 1 GGZ Friesland, Borniastraat 34b, Leeuwarden, 8934 AD the Netherlands; 2 Department of Psychiatry and EMGO Institute, Vrije University Medical Center/GGZinGeest, Amsterdam, the Netherlands; 3 Department of Research, GGZinGeest, Amsterdam, the Netherlands

**Keywords:** Clinical course, Trauma related disorders, Personality disorders, Comorbidity, Prediction

## Abstract

**Background:**

Trauma-related disorders and personality disorders are prevalent in survivors of chronic childhood trauma and neglect. Both conditions have serious consequences for patients, their families, society and public health and a high risk of development of chronicity. However, information on the long term course trajectories is lacking and predictors of course outcome in survivors of chronic childhood traumatization are unknown. The first aim of the current study is to identify two-year course trajectories of pathology in patients with trauma-related disorders and personality disorders. The second aim is to examine predictors of the course, including demographics, clinical characteristics and comorbidities.

**Methods/design:**

The study is a naturalistic two-year follow-up of 150 patients consecutively admitted to the trauma treatment program and the personality disorder treatment program respectively at GGZ Friesland, a regular Dutch mental health care center. The only exclusion criterion is insufficient mastery of the Dutch language. Participants will be assessed after 2 years of treatment through measures that have been completed at baseline, including structured clinical interviews to measure childhood histories of trauma and neglect, (symptoms of) trauma-related disorders and personality disorders, and psychological questionnaire measures (e.g., general psychopathology, depressive symptoms and personality features). In addition, participants will complete an evaluation questionnaire to assess medication prescribed and treatment (s) received outside GGZ Friesland between baseline and follow-up. Information about (psychological and pharmacological) treatment received at GGZ Friesland during the follow-up period will be collected from patient files.

**Discussion:**

This study provides insight in the two-year course of (comorbid) trauma-related disorders and personality disorders. Identifying predictors of the course of trauma-related and personality disorders will allow to differentiate clinical profiles and will offer indicators for treatment.

## Background

Personality disorders and trauma-related disorders are associated with significant personal and societal burden, largely because of the development of chronicity and public health consequences [1, 2]. Though prevalent and impairing conditions, data on the long-term prognosis (course) of trauma-related disorders and personality disorders are scarce, in particular among survivors of chronic childhood traumatization. The available data suggest that both groups of disorders tend to run a chronic course. However, most studies tend to focus on the naturalistic short-term course of the disorders and do not focus on the (course of the) comorbidity between both categories of disorders, e.g. [3–5].

With regard to trauma-related disorders, there are several studies focusing on the natural course of posttraumatic stress symptoms or disorder (PTSD) [3, 4]. Many, if not most of these studies focusing on course of PTSD make no clear distinction between the type of trauma (simple or chronic, interpersonal or not), nor between the onset of trauma (in childhood or adulthood). A recent review concludes that trajectories of PTSD after intentional traumatic experience, i.e. deliberate infliction of harm, show wide variability: among the patients who develop PTSD, one third remit within 3 months, while in nearly 40% of cases PTSD may become chronic. This review also notices that only a few studies between 1998 and 2010 have followed participants for more than a year [5]. One of these studies [3] with a longer (i.e. 5 year) follow-up among patients with anxiety disorders shows that PTSD is a persistent illness. Another important result of this study is that trauma in childhood, compared with trauma in adulthood, predicts a longer time to remit from chronic PTSD, especially in case of comorbidity with Borderline Personality Disorder (BPD). Also, a later study found that childhood sexual abuse (CSA) predicts less improvement of PTSD symptomatology when comorbid with BPD [6]. However, a study among veterans receiving inpatient treatment for PTSD indicates that a history of childhood trauma (i.e. physical or sexual abuse, or witnessing family violence or deaths before age 7) does not predict a less favourable short-term course of PTSD symptoms, but rather the severity or complexity of the trauma-related symptoms, i.e. the presence of complex PTSD (here formulated as Disorder of Extreme Stress Not Otherwise Specified (DESNOS)) [7]. Affect dysregulation is a pervasive symptom in survivors of childhood trauma, associated with both trauma-related disorders and personality disorders [8, 9]. Although some studies did measure affect dysregulation as a symptom of complex PTSD [10], there are no studies known to specifically report data on the course of affect dysregulation.

Regarding clinical complexity, a recent meta-analysis [11] reports a comorbidity rate of any personality disorder for PTSD of 35%. This meta-analysis shows that PTSD is clinically heterogeneous, implying that, compared to all other anxiety disorders, PTSD has a highly different comorbidity profile with a mixture of personality disorder comorbidity. It suggests that the heterogeneity in clinical profile may be explained by a large variety in nature and impact of traumatic exposures. Furthermore, (cumulative) trauma during developmental years predicts increasing symptom complexity in adults, whereas adulthood trauma does not [12].

Several studies focusing on PTSD and comorbid dissociative symptoms among early traumatized patients indicate that higher levels of dissociative symptoms are accompanied by higher levels of PTSD symptoms, while the level of dissociation based on self-report (DES) does not seem to influence the course of symptoms of PTSD during treatment [10, 13]. A naturalistic 12-month post-treatment follow-up study of early traumatized inpatients with PTSD found that patients with a co-occurring complex dissociative disorder, i.e. dissociative identity disorder (DID) and dissociative disorder not otherwise specified with clinical features of DID (DD NOS-1), need more time to improve on dissociation, symptoms of PTSD, depression, general psychiatric symptoms and interpersonal functioning, compared to patients without these co-occurring disorders [14]. Recently, a study on the two-year course of post-traumatic stress symptoms and dissociative symptoms in female survivors of childhood abuse, who received 6 weeks of inpatient treatment, reports significant improvement of the global symptom load, PTSD symptoms and depressive symptoms, but no significant improvements concerning dissociative symptoms [15]. Although a group was identified which remained stable or improved during the follow-up period, no significant predictors of course outcome were found.

Study findings on the course of personality disorders suggest that even severe personality disorders can improve significantly within a couple of years, with the majority of the studies concerning BPD [16–19]. Two longitudinal studies, the Collaborative Longitudinal Personality Disorder Study (CLPS) [20, 21] and the McLean Study of Adult Development (MSAD) [22], focus on identifying features that influence the course of several personality disorders. Both studies conclude that a history of trauma or neglect predicts a more negative course of BPD. The CLPS also reports that patients with either schizotypical personality disorder or BPD had experienced higher rates of being physically attacked and reported more types of traumatic exposure. Furthermore, the CLPS finds that the severity of personality psychopathology, i.e. the number of criteria met, and a low score on the Global Assessment of Functioning (GAF), have a negative influence on the course of BPD after 2 years of treatment [20]. The MSAD, focusing on a longer (10-year) course of borderline personality disorder, finds that a younger age, no history of childhood sexual abuse, less severe childhood abuse and neglect, less severe violence witnessed as a child, no prior psychiatric hospitalization, absence of PTSD and anxious cluster personality disorders, as well as four facets of normal personality (low neuroticism and high extroversion, agreeableness and conscientiousness) predict an earlier time to remission of the BPD, when measured every 2 years [22].

**Fig. 1:**
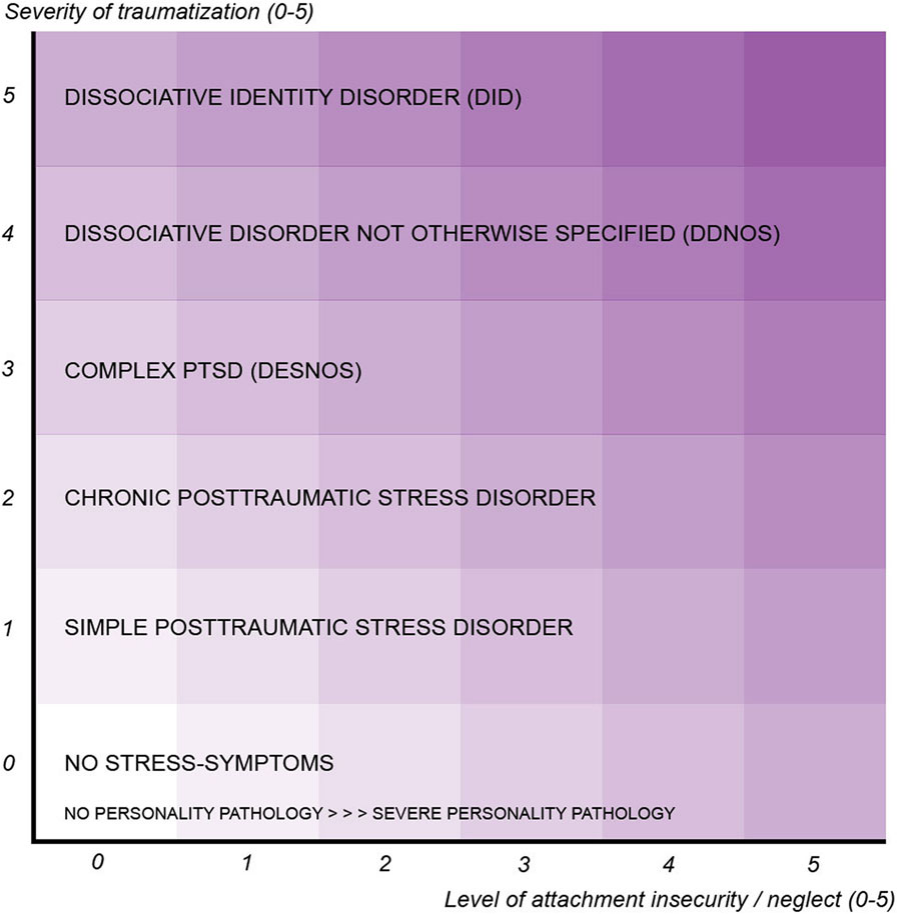
A diagnostic model for the spectrum of trauma-related disorders and personality disorders. This two-dimensional diagnostic model accounts for the influence of trauma and neglect on the development of (a spectrum of) trauma-related disorders as well as personality disorders. The severity of trauma (based on factors such as the age of onset, how frequently it occurred, the relationship to the perpetrator, and the number of perpetrators) endured, situated on the y-axis, is assumed to be related to the severity of trauma-related disorders. Situated on the x-axis, the severity of emotional neglect (the quality of the early bond with the primary caregivers) represents the second dimension. This dimension is assumed to be related to the severity of personality disorders. The numbers (0–5) on the y-axis and the x-axis represent the severity scores on both dimensions, which ranges from no trauma or emotional neglect endured (score 0) to very severe trauma or emotional neglect (score 5). Individual patients can be ‘located’ somewhere in the two-dimensional square

Although many studies have focused on describing the comorbidity between trauma, trauma-related disorders and personality disorders [23], little research has been done on the long term course of these comorbidities. This lack of information is due to methodological limitations of previous studies, like short term follow-up [7, 14], using only (self-report) symptom questionnaires [10, 13, 15], and, concerning (comorbidity of) personality disorders, focussing only on specific disorders, like BPD [19, 20]. We designed the present study to overcome these limitations of the previous studies and expect therefore to be better able to identify predictors of two-year course outcome. Furthermore, the present study will test a theoretical model of Draijer [23, 24], while previous studies lack a theoretical background. We use the diagnostic square of Draijer (see Fig. 1) to study the (comorbid) course of trauma-related disorders and personality disorders. This two-dimensional diagnostic model accounts for the influence of trauma and neglect on the development of (the spectrum of) trauma-related disorders as well as personality disorders. The severity of trauma endured, as the first dimension, situated on the y-axis, fluctuates depending on factors such as the age on which the trauma occurred, how frequently it occurred and the relationship to the perpetrator. This dimension is assumed to be related to the severity of trauma-related disorders. Situated on the x-axis, the severity of emotional neglect represents the second dimension. This dimension is assumed to be related to the severity of personality disorders. Thus a square is created, that gives an indication on the treatability of these disorders by psychotherapy, with highly treatable psychopathology on the low end of the dimensions, and highly untreatable psychopathology on the high end of the dimensions [24]. While this model already has shown its relevance in clinical practice as a diagnostic model, the aim of this study is to test if it can be validated as a prognostic model for course of illness: will it be affected by characteristics of the patient, treatment (s) received and/or (the type and severity) of the (comorbid) symptoms of trauma-related and personality disorders.

The current study is a two-year follow-up of trauma-related and personality disorders in patients who sought treatment at a regular Dutch mental health care center, GGZ Friesland. The purpose is to examine the two-year course of trauma-related disorders and personality disorders, i.e. (symptomatic) improvement of the (comorbid) disorders, as well as identifying predictors of a (non-) favourable course of these symptoms. The duration of the follow-up period is based on clinical experience in treating patients with childhood trauma and neglect. Furthermore, in determining the follow-up period, we have taken several factors into account: because trauma-related disorders and personality disorder mostly run a chronic course, a short follow-up period, e.g. 6 months or 1 year, will probably not be able to show significant changes; however to minimalize non-response, the follow-up period should not be too long, while patients are more likely to have ended their therapy, have moved, changed their telephone number et cetera.

The primary research question is: what is the course of (symptoms of) trauma-related and personality disorders in a sample of patients in treatment over a follow-up period of 2 years? More specifically, what is the course of the core symptoms (PTSD, (symptoms of) dissociative disorders, (symptoms of) personality disorders affect dysregulation and general functioning) in a sample of treatment seeking adults with and without histories of early childhood trauma, i.e. before the age of 12? The second research question is: which variables predict course outcome after 2 year? More specifically, are demographic (gender, age) and clinical (dissociation, comorbidity in general, personality features) predictors of a (un) favourable course after 2 years? And to what extent is course outcome dependent on the type or duration of treatment (e.g., inpatient, outpatient, pharmacotherapy, psychological treatment)? The specific variables and (possible) predictors are described more extensively in the method.

Based on the theoretical model of Draijer [23, 24] and results of previous studies, we expect that type and severity of personality pathology and trauma-related pathology at baseline predict the size of improvement at follow-up, with severe (comorbid) pathology predicting less improvement than light to medium (comorbid) pathology. Furthermore, we expect that type and severity of reported trauma and neglect as well as the number of reported traumatic experiences predict the level of improvement in symptoms of trauma-related or personality disorders between baseline and follow-up. Also, we expect that level of improvement in symptoms of trauma-related or personality disorders correlates with the level of improvement of other more general psychopathology, like anxiety, depression, and general functioning. Finally, we expect that dose (amount) and type of treatment can be identified as predictors of the course of symptoms of trauma-related and personality disorders.

## Methods/design

The design of this study is a naturalistic two-year follow-up of patients admitted to the trauma treatment program and the personality disorder treatment program at GGZ Friesland, the Netherlands. The study is supervised by the Department of Psychiatry, Vrije University Medical Center, Amsterdam, the Netherlands, experts in cohort studies.

### Ethical approval

The study protocol is approved by the Medical Ethics Committee of the Regionale Toetsingscommissie Patiëntgebonden Onderzoek (RTPO, registration number NL 47054.099.14).

### Participants

Patients who sought treatment at GGZ Friesland for either personality disorders or trauma-related disorders were recruited for the baseline data collection. Baseline data were collected between November 2011 and March 2014. The inclusion period of the follow-up assessment runs from February 2014 until August 2016. All patients who completed baseline assessment will be invited to participate in the follow-up study, 2 years after they completed the baseline assessment. This includes 150 patients (116 women and 34 men): 49 patients who sought treatment for traumatic experiences and were admitted to a specialized trauma-related disorders treatment program, aimed specifically at adult survivors of prolonged early childhood trauma, and 101 patients who sought treatment for personality pathology and were consecutively referred to a specialized personality disorders treatment program. For a detailed description of the inclusion at baseline, we refer to [23]. Only participants with insufficient mastery of the Dutch language were excluded. Data on non-response as well as representativeness of the baseline sample are presented elsewhere [23]. The baseline sample was considered to represent the two relevant clinical populations.

### Procedure

For follow-up, all 150 patients who participated in the baseline study will receive an information letter, which explains the background and the purpose of the study, how the collected data will be stored, guarantee of privacy and voluntary nature of the participation. The main researcher will approach patients by phone or, if they cannot be reached, by e-mail or letter; patients get the opportunity to ask questions about the study and are invited to participate. When patients agree to participate, written informed consent will be obtained and a first appointment with an interviewer scheduled. Appointments take place at a location of GGZ Friesland or, if requested, at a patient’s home. Usually it will take two or three appointments to complete the extensive assessment, depending on the patient’s preference. Patients will be asked to complete the self-report questionnaires at home. However, we offer assistance if patients have trouble filling in the questionnaires on their own, e.g. by clarifying difficult words in the questionnaires, answering questions of patients about the questionnaires or, in the case of a blind patient, reading the questions out loud. All interviewers are trained and supervised psychologists, who also conducted the assessments in the baseline collection wave. The results of follow-up assessment (present diagnosis) and the two-year course (change in symptoms between baseline and follow-up) will be communicated to the patient by the main researcher in the form of a psychological report.

### Outcome variables

Primary outcome variables are PTSD symptoms and disorder, dissociative symptoms and disorders, (symptoms of) personality disorders and symptoms of affect dysregulation. Secondary outcome variables are general psychopathology, anxiety symptoms, depressive symptoms and personality pathology.

### Predictors

To identify predictors of course outcome, demographics (gender, age) and type and duration of treatment during the follow-up period and before baseline are registered. Furthermore, dissociation and general personality features will be assessed. Also, the severity of traumatic experiences and neglect will be (re) assessed as possible predictors.

### Measurements

The assessment battery consists of five structured clinical interviews and nine self-report questionnaires. All interviews were also assessed at baseline, as well as eight out of nine questionnaires. The additional questionnaire concerns questions about received treatment outside GGZ Friesland during the follow-up period (see below).

To assess the clinical outcome variables, namely the (symptoms of) trauma-related and personality disorders respectively, in a reliable fashion, structured psychiatric interviews are used. The Clinician Administered PTSD Scale (CAPS) [25] is used to assess (symptoms of) PTSD and the Structured Interview for Disorders of Extreme Stress (SIDES) [26] to assess complex PTSD and affect dysregulation. The Structured Interview for DSM-IV Dissociative Disorders (SCID-D-R) [27] is used to assess dissociative symptoms and dissociative disorders, which includes dissociative fugue, dissociative amnesia, depersonalization disorder, dissociative identity disorder and dissociative disorder not otherwise specified. The Structured Interview for DSM Personality Disorders (SIDP-IV) [28] is used to assess (symptoms of) all DSM-IV personality disorders. All of these interviews have good to excellent psychometric properties, including test-retest reliability. At baseline, inter-rater agreement intervals for all four interviews were high (.90 to.95) [23].

The secondary clinical outcome variables include symptom questionnaires. General psychopathology will be assessed using the Symptom Checklist-90-revised (SCL-90-R) [29]. The Inventory of Depressive Symptomatology (IDS) [30] is used to assess depressive symptoms, and the Beck Anxiety Inventory (BAI) [31] to assess anxiety symptoms. Personality pathology is also measured in a dimensional way. The level of (mal) adaptive personality functioning will be assessed using the Severity Indices of Personality Problems (SIPP-118) [32], and ‘schemas’ (i.e. inner representations) using the Young Schema Questionnaire [33]. All questionnaires are well known for their good validity and reliability.

To measure the type and amount of traumatic experiences of patients in their childhood and in adult life, as a possible predictor, we use the Structured Trauma Interview (STI) [34]. This interview is specifically designed to measure negative experiences in childhood and adult life. This results in an overview of traumatic experiences as well as other adversities. Neglect, also a possible predictor, is measured by two proxy’s: the first is the Parental Bonding Instrument (PBI) [35], which examines four types of parental bonding based on two dimensions: care and overprotection (parental control). The second proxy is parental dysfunction, measured by the STI [36]. Both instruments have good validity and reliability. Dissociative symptoms, as a (possible) predictor, are measured using the Dissociative Experiences Scale (DES) [37]. General personality traits, as a possible predictor, are assessed using the Neuroticism, Extraversion and Openness Personality Inventory (NEO-PI-R) [38]. For an overview of all outcome variables, see Table 1.

**Table 1:** Overview of all measurements

Construct	Instrument	Time period	Severity measure (interval)
PTSD	CAPS [24]	Last month	Yes
Complex PTSD	SIDES [25]	Last month	Yes
Affect dysregulation	SIDES [25]	Last month	Yes
Dissociative Disorders	SCID-D-R [26]	Last year	Yes
Personality Disorders	SIDP-IV [27]	Last year	Yes
General psychopathology	SCL-90-R [28]	Last week	Yes
Depressive symptoms	IDS [29]	Last week	Yes
Anxiety symptoms	BAI [30]	Last week	Yes
Dissociative symptoms	DES [36]	Last month	Yes
Personality pathology	SIPP-118 [31]	Last 3 months	Yes
	Young Schema Questionnaire [32]	Currently	Yes
Personality traits	NEO-PI-R [37]	Currently	No
Traumatic experiences	STI [33]	Life time	Yes
Neglect	PBI [34]	0 to 12 years	Yes
	STI [35]	0 to 16 years	Yes

Furthermore, to measure possible determinants of course outcome we collect data from the patient record system of GGZ Friesland, including score on the Global Assessment of Functioning (GAF) Scale, last reported clinical diagnosis and data on the received therapy during the follow-up period. Data on therapy will consist of doses (the amount of received therapy in minutes) and will be specified in treatment program/department (trauma-related or personality or other), group versus individual therapy, days of hospitalization and the use of medication. In case of treatment outside GGZ Friesland, patients are asked to fill in an additional form. This form contains questions on type and duration of treatment (s) received outside GGZ Friesland, number and duration of hospitalization outside GGZ Friesland and the use of medication (e.g. type, dose and duration), prescribed outside GGZ Friesland, in the two-year period after the baseline assessment. This information will be comparable to the data on therapy inside GGZ Friesland. In addition, data on treatment history before baseline is assessed with the STI [34].

### Power calculation

The sample size was settled to permit a logistic regression analysis for the dichotomous primary outcome measure “remission of PTSD after two year” being regressed on three predictors: 1) duration of treatment, 2) severity of traumatic experiences, and 3) neglect. We determined the sample size by application of the rule of having ten events per variable, which is a conservative rule [39]. We expect that that the percentage of remitted patients will be in the range of 25%–75%, implying 40 observations per predictor. Studying three predictors, the rule implies a minimal sample size of 120. A further assumption of a drop-out rate of 20%, made us aim at 150 participants at baseline.

### Data analysis

Characteristics of the study sample will be described, using frequencies of participants and non-participants after two-year follow-up. Possible (selective) differences between the completers and non-completers will be tested using chi-square test for categorical variables and t-test for continuous variables. The course of trauma-related and personality disorders will be determined by comparing the two-year follow-up outcome with the baseline diagnostic status on four variables. For the categorical variables, i.e. presence of PTSD diagnosis (yes/no) and presence of a personality disorder (yes/no), we use chi-square statistics. For the continuous variables, i.e. severity score on the CAPS for PTSD (range 0–144) and total number of criteria met for personality disorders et cetera, we use analysis of variance. We will use a Bonferroni correction for multiple testing.

The aim of the current study is to validate the model of Draijer as a prognostic model. For an extensive description of this model and the validation of the model as a diagnostic instrument, we refer to [23]. In short, two severity indexes will be constructed, one for traumatization and one for neglect, to test relationship between the severity of chronic childhood trauma and neglect, and the severity of respectively trauma-related disorders and personality disorders. We test the possibility of relating the diagnostic square to changeability of psychopathology within a period of 2 year, by using a changeability score, based on the sum of the two severity indexes at baseline (range 0–10), as a predictor in a regression analysis on the raw difference scores (i.e. CAPS-score at follow-up minus CAPS-score at baseline). In addition, the changeability score can be replaced by separate scores on severity of traumatization and severity of neglect, so we can compare the regression coefficients and test if both axis of the square are equally related to the observed change after 2 years.

Secondly, to find determinants (predictors) of the two-year course, we will use logistic regression analysis for dichotomous primary outcome measures, as descripted in the power calculation. Again, we will use a Bonferroni correction for multiple testing. The amount of predictors we will test, will be based on literature and the distribution of our data.

## Discussion

As with most follow-up (longitudinal) studies, the biggest challenge of this study is to reach all patients who participated in the baseline study [23]. Response rates in follow-up studies vary from 58% to 94% [2, 3, 14, 15, 22]. While the treatment of (complex) trauma-related disorders and personality disorders tend to take a long time, chances of patients still having therapy within GGZ Friesland are rather high. This makes it easier to contact patients and lowers the risk of expired telephone numbers and old addresses. However, while at baseline assessment was part of daily clinical routine for patients referred to trauma-related disorders program, the follow-up assessment is not embedded in any particular treatment program. This could lower the response rates. We expect non-response due to patients who are not able to participate (due to i.e. emigration, physical illness or death by suicide), patients who are not willing to participate (due to i.e. lack of time or motivation) and patients which cannot be reached (due to i.e. changed telephone numbers, changed addresses). To minimalize non-response during the study period, we keep in close touch with the treatment departments and treating clinicians. Furthermore, patients get well informed, we create a pleasant atmosphere during assessment and we concern (and anticipate on) the patients’ needs, as far as possible. All risks considered, we expect a non-response rate of 20%, leaving an N of 120. This N provides enough statistical power to answer the research questions.

Using the same psychologists to conduct the assessments at baseline and follow-up has both advantages and disadvantages. Psychologists must be well trained and have clinical expertise to obtain the clinical interviews in a reliable way. Psychologists meeting those criteria are scarce. The inter-rated agreement intervals were high at baseline (.90 to.95). Using other psychologist at follow-up can lower the inter-rated agreement. On the other hand, using the same psychologist can result in a bias towards a favourable course. However, we do not expect solitary favourable courses in our hypothesis. To minimalize any form of bias, psychologists are not allowed to look-up (individual) outcome at baseline, before they conduct follow-up assessment. Furthermore, we provide personal supervision by a senior clinical psychologist and encourage to reason the scoring of clinical interviews to enhance objectivity.

We overcome limitations of previous studies, as discussed above, by using structured clinical interviews as well as symptom questionnaires, carefully registering onset and type of trauma (at baseline and follow-up) and collecting several data, which potentially act as predictors of course of disorders and symptoms. Furthermore, to keep track of factors that may affect symptom change, we enumerate use of medication, received therapy (in- and outside GGZ Friesland) and traumatic experiences in the two-year period after baseline assessment. Also, we keep track of developments, like changes in offered therapies in the specialized treatment programs (of trauma-related and personality disorders) at GGZ Friesland. However, we cannot overcome some limitations, inherent in this type of research, like the use of retrospective data. Also, in addition to the clinical interviews, we use some self-report questionnaires.

An important strength of this study is that it is unique in the elaborate way in which traumatization and (personality) pathology are investigated [23]. This study will provide insight in the relation between symptoms of trauma-related disorders and personality disorders and how they affect each other in the course of treatment, while they are frequently treated as separate conditions nowadays. Identifying factors, which influence the course of the trauma-related and personality disorders, allows making predictions about the future course and offers indicators for treatment. Furthermore, the study can contribute to improve diagnosing trauma-related disorders and personality disorders as well as provide knowledge of prognostic variables that may allow the prognosis of an individual patient and the pre-classification/stratification of patients in outcome studies.

## Abbreviations

ANCOVA: Analysis of Covariance
BAI: Becks Anxiety Inventory
BPD: Borderline Personality Disorder
CAPS: Clinician Administered PTSD Scale
CLPS: Collaborative Longitudinal Personality disorders Study
DES: Dissociative Experience Scale
DESNOS: Disorders of Extreme Stress Not Otherwise Specified
DID: Dissociative Identity Disorder
DSM-IV: Diagnostic and Statistical Manual of Mental Disorders
GAF: Global Assessment of Functioning
IDS: Inventory Depression Scale
MSAD: McLean Study of Adult Development
NEO-PI-R: Neuroticism, Extraversion and Openness Personality Inventory - Revised
PBI: Parental Bonding Instrument
PTSD: Post Traumatic Stress Disorder
RCT: Randomized control trial
SCID-D: Structured Clinical Interview for DSM-IV Dissociative Disorders
SCL-90-R: Symptom Check List Revised
SIDES: Structured Interview for Disorders of Extreme Stress
SIDP-IV: Structured Interview for Personality Disorders
SIPP-118: Severity Indices of Personality Problems
STI: Structured Trauma Interview
